# The Treatment of Paralysis of Motion

**Published:** 1860-05

**Authors:** Charles F. Taylor


*The Treatment of Paralysis of Motion.* By CHARLES F. TAYLOR, M.D.

In the MEDICAL MONTHLY for November, 1858, in an article under the above title, I pointed out the method to be pursued in order to re-establish the control of the will over the muscles when this control has been suspended by any cause capable of interrupting the conducting power of the nervous tracts. I showed how, either by a process of exclusion—that is, by excluding from the conditions under which an effort is made, all tendency to movements other than the designated ones—the will could be most efficiently brought to bear on the paralyzed muscles; or by a process of concentration—that is, by causing such a position to be assumed, muscular effort will increase towards the paralyzed parts, accumulating, as it were, in these parts; and in either case, at the same time avoiding the expenditure of nervous or muscular power upon the non-affected parts, which are always most easily stimulated; and when allowed to act, (as in ordinary exercise,) absorb, as it were, the whole nervous force into themselves, and thus virtually rob the paralyzed muscles of their fair share of stimulus; and that by continuing thus, carefully to make every motion intended to be curative, following it up, day after day, the conducting power of the nerves and the contraction of the muscles, would be manifested, even where neither were perceptible at first; and that, ultimately, a large amount of the lost function might, in many cases, be regained, as illustrated by the cases presented. Subsequent experience has confirmed every position assumed in that article, and has also added some valuable ideas, which is the object of this brief paper to present. In order to make the point more clear, the following case is related:

In December, 1858, Master D. F., aged ten years, was brought to me from the country, with hemiplegia of the left side. Five years before, that is, when he was five years old, he was kicked by a horse, in the right fronto-parietal region, detaching a portion of the skull about two by three inches large, depressing and forcing it under the adjacent parts, and considerably lacerating the brain-substance. The shattered bone was removed with difficulty, and after twenty-four hours, when consciousness returned, he was found to be paralyzed in the left side. It was nearly a year before the wound had healed, during which time, according to the history of the case, a "fungus" (?) was removed from, and an abscess had formed on, the wound. At the end of a year he was well, except the paralysis, which remained nearly unaltered till I saw him, but for the past year, was rather growing worse. He had no use of the left arm, could only move it imperfectly at the shoulder and elbow; walked with difficulty, and could not support more than one-fourth or one-fifth of the weight of the body on the left leg. There was imperfect development over the whole left side; the chest was sunken on that side, and the muscles soft and small.

After treatment of three months, (unhappily stopped, at that time, from an attack of gastritis,) there was a remarkable increase of strength, and of voluntary control over the muscles, so that he could stand on the left foot for fifteen minutes; could climb a ladder, using both hands; and there was evident increase of muscular development through the whole side.

Just one year from the time he commenced, namely, in December, 1859, he resumed his treatment at my office. The development which had begun the year before, had kept steadily on. The chest and shoulders were symmetrical, so that the wadding before placed on the left side of his coat to make the shoulders appear even, had to be entirely removed; and he was stronger throughout that side. But here comes the most interesting part, so far as treatment is concerned. I observed in his case what I have observed in many other cases, that while the muscles might be brought under the *control* of the will, while they would act correctly and strongly, they would not act *readily*. In this case, though the muscles of the left leg were strong and would move as wished, they would act only tardily.

He had muscular strength enough to walk without limping, yet he limped. When he stepped with that foot, there seemed to be an interval of time after the effort before the action. The leg would partially give way for a moment, under the weight of the body, before the muscles would contract so as to sustain him firmly upon it. And yet, when this contraction did take place, it was sufficiently powerful. Hence the conclusion, that in the physiology of muscular motion there is a difference between certainty and readiness of action.

It occurred to me, that as we can develop certainty of action by concentrating the will upon a part and *prolonging* this effort, we might secure rapidity of action by *sudden* efforts thus concentrated. I therefore adopted a system of movements involving sudden *explosions* (as it were) of effort upon the affected parts. This process was kept up for two months, with more than anticipated results. He gained nothing in strength or certainty of movement, but in readiness and rapidity of muscular action there was as much improvement as there had been before in control of it. He can now walk with scarcely a perceptible difference in quality of movement in the two sides.

Muscular contraction follows immediately upon the effort, so that the settling down of the left side in walking, before so marked, is scarcely perceptible.

The principle of distinguishing between the different qualities of force and the peculiar kind of functional manifestation to be employed in order to secure it, has been applied in many other cases with uniformly favorable results. Another important matter in the treatment of paralysis is, to secure a good circulation of blood in the affected parts before attempting to reach voluntary motion in them. Innervation as well as muscular contraction take place only in the presence of arterial blood. Before the patient essays to move a paralyzed limb, especially at the beginning of treatment, the muscles should be stretched, (passively,) or made to act through position and reflex action, (as standing on a paralyzed leg, for instance, while held in position by assistants;) and one is often surprised to notice how readily the previously rigid muscles will move in obedience to a volition.

Out of over forty cases which I have treated during the last three years, more than three-fourths have shown marked improvement; but the most favorable of all have been those unfortunate cases of "withered limbs" in children, occurring from teething, (inanition,) fevers, falls, &c., when quite young.

In a subsequent communication I intend to relate a number of these cases, which, to me, exceed in interest all others combined; especially when we consider the effect of a cure on the child's future prospects in life.

29 *Cooper Institute, New York.*

